# A Novel Laboratory-Scale Mesocosm Setup to Study Methane Emission Mitigation by *Sphagnum* Mosses and Associated Methanotrophs

**DOI:** 10.3389/fmicb.2021.651103

**Published:** 2021-04-26

**Authors:** Martine A. R. Kox, Alfons J. P. Smolders, Daan R. Speth, Leon P. M. Lamers, Huub J. M. Op den Camp, Mike S. M. Jetten, Maartje A. H. J. van Kessel

**Affiliations:** ^1^Department of Microbiology, IWWR, Radboud University, Nijmegen, Netherlands; ^2^Department of Aquatic Ecology and Environmental Biology, IWWR, Radboud University, Nijmegen, Netherlands; ^3^B-WARE Research Centre, Nijmegen, Netherlands

**Keywords:** methanotrophy, peatland restoration, *Sphagnum* moss, methane cycle, mesocosm

## Abstract

Degraded peatlands are often rewetted to prevent oxidation of the peat, which reduces CO_2_ emission. However, the created anoxic conditions will boost methane (CH_4_) production and thus emission. Here, we show that submerged *Sphagnum* peat mosses in rewetted-submerged peatlands can reduce CH_4_ emission from peatlands with 93%. We were able to mimic the field situation in the laboratory by using a novel mesocosm set-up. By combining these with 16S rRNA gene amplicon sequencing and qPCR analysis of the *pmoA* and *mmoX* genes, we showed that submerged *Sphagnum* mosses act as a niche for CH_4_ oxidizing bacteria. The tight association between *Sphagnum* peat mosses and methane oxidizing bacteria (MOB) significantly reduces CH_4_ emissions by peatlands and can be studied in more detail in the mesocosm setup developed in this study.

## Introduction

Globally, about 15% of peatland area has been drained for agriculture, forestry or bioenergy production, with highest losses in Europe (Joosten and Clarke, 2002; Grootjans et al., 2012). Drainage results in the exposure of the organic peat layer to oxygen, resulting in high CO_2_ emission (Waddington and Day, 2007; Abdalla et al., 2016; Reumer et al., 2018). As restoration measure, drained peatlands can be rewetted to protect organic matter from fast aerobic degradation (Grootjans et al., 2012; Renou-Wilson et al., 2019). However, the resulting anaerobic conditions create a suitable environment for the production of the potent greenhouse gas methane (CH_4_), leading to high methane emissions (Harpenslager et al., 2015; Abdalla et al., 2016; Renou-Wilson et al., 2019). The water table is a well-known factor in controlling CH_4_ cycling in wetlands (Bridgham et al., 2013; Ho et al., 2016); when the water table remains below the field surface, CH_4_ emissions typically remain low. However, when the water table rises, the oxygen concentration decreases which results in a strong increase in CH_4_ emission (Smolders et al., 2003; Harpenslager et al., 2015).

The CH_4_ emission of rewetted peatlands seems to be strongly reduced by the presence of (aquatic) *Sphagnum* mosses, which harbor CH_4_-oxidizing microorganisms that consume the produced CH_4_ (Smolders et al., 2003; Raghoebarsing et al., 2005; Waddington and Day, 2007; Kip et al., 2010). The association between these microorganisms and *Sphagnum* mosses was shown to be mutually beneficial (Raghoebarsing et al., 2005). By producing CO_2_, methanotrophs can relieve the CO_2_ limitation that *Sphagnum* mosses experience in submerged conditions (Smolders et al., 2001; Raghoebarsing et al., 2005; Kip et al., 2010). In return, MOB can benefit from O_2_ production and shelter provided by the *Sphagnum* moss (Kip et al., 2010). Different types of MOB are associated to *Sphagnum* mosses. Molecular surveys showed that *Alphaproteobacterial* methanotrophs typically dominate 16S rRNA gene-libraries from *Sphagnum* mosses (Kip et al., 2011; Kox et al., 2016, 2018). Within the *Alphaproteobacteria* especially methanotrophs of the family *Methylocystaceae* (*Methylocystis spp.*) and the acidophilic methanotrophs of the family *Beijerinckiaceae* (*Methylocella, Methyloferula*, and *Methylocapsa*) are often found and several of these have been isolated from peatlands (Dedysh et al., 2000, 2004; Dedysh, 2011; Vorobev et al., 2011; Danilova et al., 2013). In addition, it has been shown that also gammaproteobacterial methanotrophs are present and active in peatlands (Kip et al., 2010; Esson et al., 2016; Kaupper et al., 2021) with species belonging to *Methylomonas*, *Methylobacter, Methylovulum*, and *Methylomicrobium* being described as active members.

**FIGURE 1:**
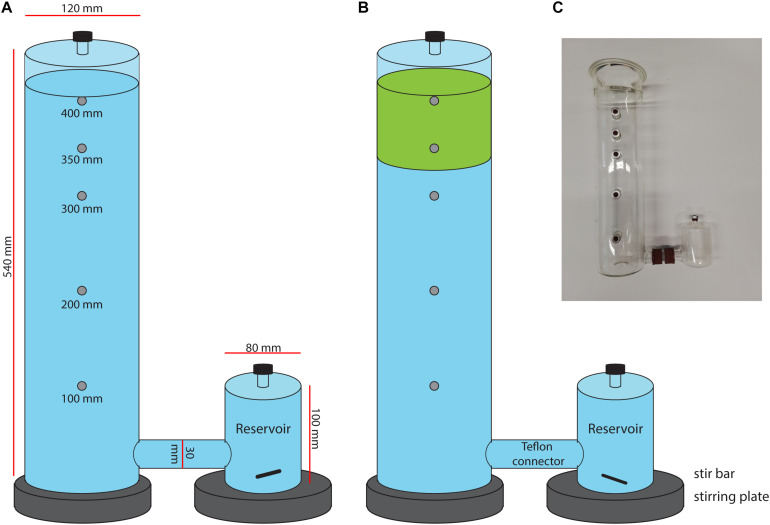
Schematic set up of mesocosm incubation with in **(A)** control mesocosms containing only filtered peat water (blue) and in **(B)** the moss mesocosms, containing *Sphagnum* moss layer (green) in filtered peat water. **(C)** Picture of the mesocosm setup.

Peatland methane fluxes have been studied in the field, but it is challenging to investigate the association between methanotrophs with *Sphagnum* mosses in more detail. Therefore, investigating methanotroph-Sphagnum interactions and methane-flux dynamics under laboratory-controlled conditions can provide detailed insights into underlying mechanisms. The goal of our study was to establish the role of *Sphagnum* mosses and associated methanotrophs in mitigating CH_4_ fluxes from rewetted peatlands. An excellent case study for a rewetted peatland is the Mariapeel peatland in Netherlands, which has been drained since 1998. The peatland was rewetted for restoration purposes, which resulted in a decrease in CO_2_ emission, but a strong increase in CH_4_ emission (Smolders et al., 2003). We developed a new mesocosm set-up (Figure 1) in which methane-oxidation by submerged *Sphagnum* mosses can be studied in detail in a controlled laboratory setup, without the variability encountered in the field. We hypothesized that the submerged *Sphagnum* moss layer acts as a biofilter for CH_4_, thereby reducing CH_4_-emission to the atmosphere. Furthermore, it was expected that CH_4_-oxidizing microorganisms are associated with *Sphagnum* mosses, rather than with peat water. Monitoring of the CH_4_-flux throughout the mesocosm incubation, as well as CH_4_ batch-assays and molecular analysis of 16S rRNA and methane monooxygenases (*pmoA* and *mmoX*) genes, showed that methanotrophs were highly active and enriched in the newly developed mesocosm setup.

## Materials and Methods

### Sampling Site and Field Measurements

The sampling site was located in the Mariapeel (51°24′28.4″N, 5°55′8″E), a peat bog nature conservation area in the southeast of the Netherlands. This site was visited for measurements and sampling in August 2017. Net diffusive gas fluxes were measured in the field using a fast greenhouse gas analyzer with cavity ringdown spectroscopy (GGA-24EP; Los Gatos Research, United States) connected to a Perspex chamber (15 cm in diameter). The chamber was put on top of the moss for 10 min to measure fluxes of CO_2_ and CH_4_. In total three independent measurements were taken within two meter distance from each other. After removal of the moss layer and an equilibration period of 15 min, measurements were repeated. Submerged *Sphagnum cuspidatum* moss and water was collected after the measurements. Upon arrival in the laboratory, all samples were stored at 4°C until the start of the incubations. The experiment was performed twice; for the first mesocosm experiment, mosses were stored 1 day. For the second mesocosm experiment, this was 35 days.

### Mesocosm Design

The mesocosm consists of a glass cylinder with a diameter of 12 cm and a height of 54 cm, with a separate reservoir that was connected with a Teflon connector (see Figure 1 and Supplementary Figure 1). The reservoir volume was 0.5 L, the connector tube volume 0.1 L and the column volume 5.5 L. The liquid level in the mesocosms was maintained at 5.1 L. The column headspace was closed using a greased lid with a sampling port. Several sampling ports (in the reservoir, cylinder headspace and in the cylinder at 10, 20, 30, 35, and 40 cm height) allow for sampling at different heights. Sampling ports were closed using red butyl rubber stoppers and aluminum crimp caps.

### Mesocosm Incubation

The mesocosm incubations were performed in duplicate for 32 days at room temperature. Two mesocosms were incubated simultaneously, one containing 100 *Sphagnum cuspidatum* plants (6 cm in length each, 120 g fresh weight in total) in filtered peat water and one contained only filtered peat water. Prior to incubation, *Sphagnum* mosses were carefully rinsed with tap water. Both mesocosms had an acclimatization period of 7 days prior to sampling. CH_4_ was added via the enclosed reservoir, which was stirred with a 2 cm magnetic stir bar at 250 rpm. Mesocosms were opened for 1 h per day to allow aeration. Directly after aeration, 20 ml of CH_4_ and 5 ml CO_2_ was injected in the reservoir. Light (16 h light, 8 h dark) was supplied on top of the mesocosm column using 120 deep red/white LEDs (Philips, Green-Power LED, Poland; 150 μmol m^–2^ s^–1^ photosynthetically active radiation at vegetation level). As additional control incubation, 100 plantlets of gnotobiotic *Sphagnum fimbriatum* (obtained from moss stock center Freiburg, Germany) were incubated.

### Mesocosm CH_4_ Fluxes

CH_4_ concentrations were measured directly after closing (0 h) and just before opening (23 h) the mesocosms by collecting 0.5 ml gas or water samples which were injected into a closed 5.9 ml Exetainer vial (Labco Ltd., Lampeter, United Kingdom). The concentration of dissolved CH_4_ throughout the column was determined once a week, by sampling water at four different time points during the day (0 h, 3 h, 7 h, and 23 h after closing the headspace). The CH_4_ concentration in the Exetainers (Labco Ltd., Lampeter, United Kingdom) was measured at least 4 h after sampling by using a gas chromatograph with a flame-ionized detector and a Porapak Q column de Jong et al. (2018). Dissolved CH_4_ in the water was calculated based on the solubility of CH_4_ and was accounted for in the flux calculations as well. The daily CH_4_ flux in the mesocosm was calculated as the change in CH_4_ concentration in the headspace, divided by the surface area (0.01131 m^2^).

### Potential CH_4_ Oxidation Rates

Prior to and after the mesocosm incubation, moss (3 g fresh weight) and peat water (12 ml, unfiltered) and filtered (0.2 μm) was incubated in 120 ml serum vials that were closed with red-butyl rubber stoppers and metal crimp-caps. Each serum vial received 2 ml CH_4_ (1.8%) and CH_4_ concentration was measured as described above. As a control for biological methane oxidation, 6 ml of acetylene was added after 10 h of incubation with CH_4_.

### Water Geochemistry

Water geochemistry was measured for both unfiltered and filtered (2–5 nm pore size, HF80S dialysis filter, Fresenius Medical Care, Homburg, Germany) peat water (see Supplementary Table 9). The pH was measured and elemental composition was determined using ICP-OES as described previously (Kox et al., 2018).

### DNA Extraction

Five grams of moss (fresh weight) was taken from the mesocosm incubations and directly grinded using a pestle and mortar and liquid nitrogen, after which DNA was extracted using the DNeasy PowerSoil DNA extraction kit following manufacturers protocol (Qiagen Benelux B.V., Venlo, Netherlands). DNA quality was checked by gel-electrophoresis and using the Qbit dsDNA HS Assay Kit (Invitrogen, Thermo Fisher Scientific, Carlsbad, CA, United States).

### Amplicon Sequencing and Analysis

Barcoded Amplicon-sequencing of the amplified V3–V4 region of the bacterial 16s rRNA gene [primers Bact-341f and Bact 785r (Klindworth et al., 2013)] was performed by BaseClear B.V. (Leiden, Netherlands) using Illumina Miseq. The obtained 326045 reads were quality filtered and analyzed using Mothur (v1.36.1), following the Illumina Standard Operating Procedure (SOP, accessed on May 8th 2018, Kozich et al., 2013). Merged reads shorter than 400 bp were discarded, chimeras were removed using the UCHIME algorithm (Edgar et al., 2011) and the remaining sequences were clustered at 97% identity. The resulting OTUs were classified based on the SILVA v132 16s rRNA gene non-redundant database (SSURef_99_v132_SILVA). Non-target sequences (Chloroplasts, Mitochondria, unknown, Archaea, and Eukaryota) were removed from the dataset (see Supplementary Tables 1, 2 for full overview of read processing). The output was analyzed with R [version 3.4.0 by the R Development Core Team (2017)] and Rstudio v1.1.456 (RStudio Team, 2016) using the packages Phyloseq (McMurdie and Holmes, 2013) and Vegan (Oksanen et al., 2016). Singletons were removed, and read libraries of all samples were rarefied by random subsampling (seed: 12345) to 6500 reads per sample (Rarefaction curves are depicted in Supplementary Figure 2). As a follow-up a PcoA plot (Supplementary Figure 4) was created using Phyloseq, based on a Bray-Curtis dissimilarity matrix on rarefied data. All sequencing data can be accessed via GenBank NCBI BioProject PRJNA517391.

### Quantitative PCR

Copy numbers of the Bacterial 16S rRNA gene and *pmoA* and *mmoX* genes were quantified by qPCR (see Supplementary Table 3 for primers). The qPCR reaction mix consisted of PerfeCTA Quanta master mix (Quanta Biosciences, Beverly, MA, United States) and 0.5 ng sample DNA and 1 μl of each primer (10 μM). qPCR reactions were performed in triplicate with a C1000 Touch thermal cycler equipped with a CFX96 Touch^TM^ Real-Time PCR Detection System (Bio-Rad Laboratories B.V., Veenendaal, Netherlands). Triplicate measurements per sample were averaged prior to statistical analysis. Standard curves were obtained via 10-fold dilution series of a PGEM T-easy plasmid (Promega, Madison, WI, United States) containing the target gene. Data was analyzed using Bio-Rad CFX Manager version 3.0 (Bio-Rad Laboratories B.V., Veenendaal, Netherlands).

### Statistics

Statistics were performed by using R version 3.4.0 by the R Development Core Team (2017). In order to allow for parametrical statistical tests, Shapiro–Wilk’s test was used on the residual (stats-package) to test the normality of the data and Levene’s test (car-package) was used to test for homogeneity of variance. If assumptions of tests were not met, data was log-transformed (ln), which was the case for the field CH_4_ flux data. A paired *T*-test was used to test whether the net CH_4_ flux in the field was affected by the presence of moss (moss field/moss removed). Differences in the potential CH_4_ oxidation activity in peat water and mosses prior to mesocosm incubation were tested using a non-parametric Kruskal Wallis tests. Within each material (moss/peat water) the effect of treatment (field/washing or filtering) was tested using an independent *T*-test. Differences in the potential CH_4_ oxidation activity after mesocosm incubation were tested using a 3-way ANOVA, followed by a Tukey HSD *post hoc* test. Differences in copy number between each moss sample for each target gene were analyzed using a one-way ANOVA, followed by a Tukey HSD *post hoc* test. Here, the data for *16S rRNA* gene and *mmoX* gene were log-transformed (ln) prior to analysis.

## Results

### Field CH_4_ Flux

**FIGURE 2:**
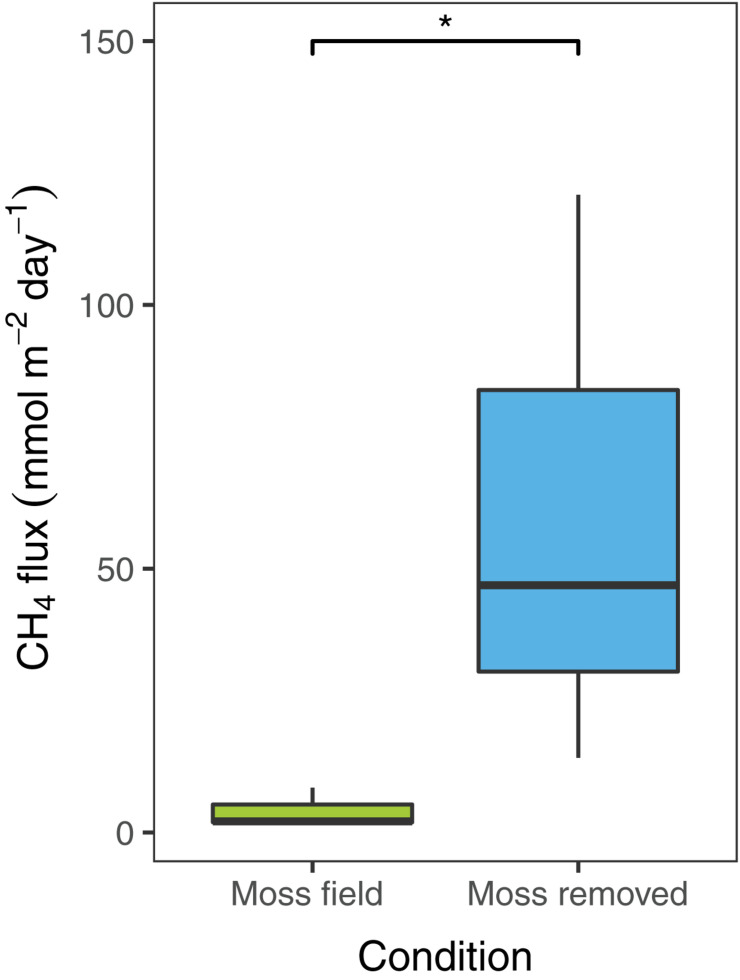
Net CH_4_ flux (mmol CH_4_ m^– 2^ day^– 1^) measured in the field with *Sphagnum* moss layer present (green, *n* = 3) and after moss removal (blue, *n* = 3). Error bars indicate the standard error of the mean, an asterisks indicates *p* < 0.05.

To estimate diffusive CH_4_ emissions in the field, flux-chamber measurements were conducted in plots with submerged *Sphagnum* mosses before and after removal of the moss layer. The CH_4_ emission in the field situation with the submerged *Sphagnum* moss layer was 4.1 ± 2.1 mmol CH_4_ m^–2^ day^–1^ (mean ± SEM, *n* = 3; Figure 2). Removal of the *Sphagnum* moss layer significantly increased the net CH_4_ emission [*t*_(__2__)_ = −6.1, *p* < 0.05] to 60 ± 32 mmol CH_4_ m^–2^ day^–1^ (Figure 2).

### CH_4_ Oxidation Activity Prior to Mesocosm Incubation

**FIGURE 3:**
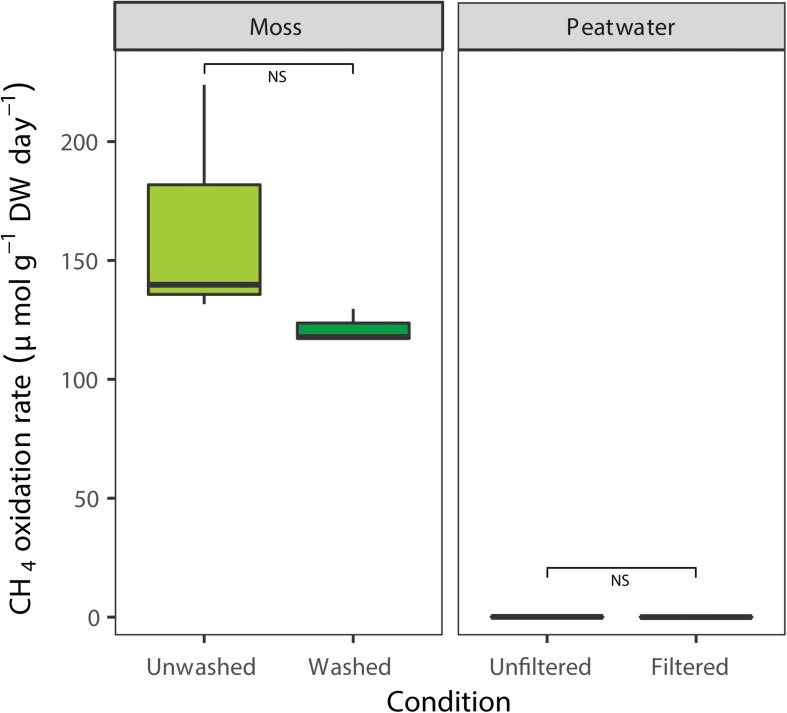
Potential CH_4_ oxidation rate in batch, associated with field *Sphagnum* mosses (light green, μmol CH_4_ g^– 1^ DW day^– 1^) or washed *Sphagnum* mosses (darker colors) and rates in peat water unfiltered or filtered. Error bars indicate the standard error of the mean (*n* = 3).

The CH_4_ oxidation rates associated with the *Sphagnum* moss and peat water were determined prior to incubation in the mesocosm setup by using batch assays (Figure 3). Methane oxidation was clearly associated to *Sphagnum* mosses, which showed higher CH_4_ oxidation rates (average rate mosses 143 ± 17 μmol g DW^–1^ day^–1^, Figure 3) compared to peat water, which had virtually no activity (0.05 ± 0.06 μmol g DW^–1^ day^–1^; χ^2^ = 7.5, *p* < 0.01, Supplementary Figure 5 and Supplementary Table 6). Washing of *Sphagnum* mosses reduced the CH_4_ oxidation rate by 15% to an average CH_4_ oxidation rate of 121 μmol g DW^–1^ day^–1^; [*t*_(__2__)_ = 1.5, *p* > 0.05, Figure 3], indicating that most MOB were strongly associated with the mosses.

### Methane Emission by the Mesocosm Incubations

**FIGURE 4:**
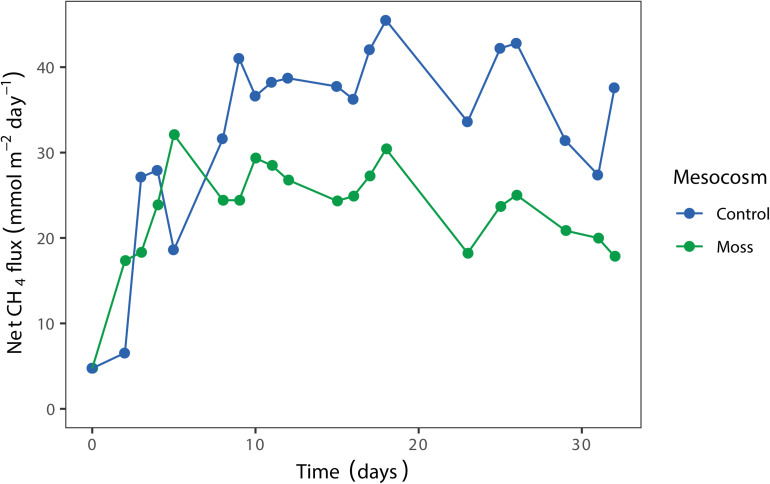
Net CH_4_ flux (mmol CH_4_ m^– 2^ day^– 1^) from the mesocosms with *Sphagnum* moss (green) and control mesocosm with only peat water (blue), measured in the headspace over time (days). Each dot represents the mean of two technical replicates.

The net CH_4_ flux in the mesocosm showed a similar pattern for moss containing and control mesocosm until day 8 after incubation (Figure 4). Thereafter, the CH_4_ concentration in the headspace of the *Sphagnum* moss containing mesocosm was lower compared to the control mesocosm. In addition, the CH_4_ emission from the *Sphagnum* moss mesocosm gradually decreased during the 32 days incubation period, which is a strong indication of increasing CH_4_ oxidation activity. A second replicate of the experiment showed a similar pattern, with lower CH_4_ emission when *Sphagnum* mosses were present in the mesocosm (Supplementary Figure 8 and Supplementary Tables 7, 8). In order to test if the methane emission is decreased by activity of microorganisms associated to the moss or by a decreased diffusion of methane from the liquid to the gas phase of the column; a similar experimental setup was performed using *Sphagnum* mosses without any associated microorganisms (gnotobionts). Net CH_4_ flux from this mesocosm was about 20% lower than the moss control (Supplementary Figure 9), indicating that both a decreased diffusion rate and microbial activity play a role in the observed decrease in CH_4_ emission.

### CH_4_ Oxidation Activity After Mesocosm Incubation

After 32 days of incubation in the mesocosms, *Sphagnum* moss and peat water were taken from the mesocosm in order to measure potential CH_4_ oxidation activity in batch. The activity of the mosses was 189 μmol CH_4_ g^–1^ DW day^–1^ (Table 1). Even after 32 days of incubation, peat water showed no CH_4_ oxidation activity (*R*^2^ < 0.9; see Table 1 and Supplementary Figures 6, 7), again indicating a tight association of the CH_4_ oxidizing microorganisms with the moss. CH_4_ oxidation associated with mosses was almost completely inhibited by acetylene [*F*_(__1_,_4__)_ = 981.3, *p* < 0.001, Table 1], indicating that the CH_4_ oxidation is indeed entirely performed by methanotrophic microorganisms associated to the moss. During the incubation, the CH_4_ oxidation activity associated to the moss had increased by 155% (from 121 to 189 μmol g DW^–1^ day^–1^; Table 1 and Figure 3).

**TABLE 1:** Potential CH_4_ oxidation (PMO) rate in batch, after mesocosm incubation.

Material	Mesocosm	Treatment	Potential methane oxidation rate		SEM	*R*^2^	*n*
			(μmol CH_4_ g^–1^ DW day^–1^)				
**Moss**	Moss		189	*a*	6	0.98	3
**Moss**	Moss	+ acetylene	2.0	*b*	2	0.3	3

**Material**	**Mesocosm**	**Treatment**	**Potential methane oxidation rate**				
			**(μmol CH_4_ ml^–1^ day^–1^)**		**SEM**	***R*^2^**	***n***

**Water**	Moss		0.02	*a*	0.02	0.17	3
**Water**	Moss	+ acetylene	0.03	*a*	0.01	0.51	3
**Water**	Peat water only		0.09	*b*	0.01	0.86	3
**Water**	Peat water only	+ acetylene	0.05	*b*	0.01	0.67	3

*Moss and peat water samples from each mesocosm were incubated in batch, with or without acetylene. Different italic letters indicate statistical differences between PMO rates, tested by 3-way ANOVA.*

### *Sphagnum* Associated Microorganisms

**FIGURE 5:**
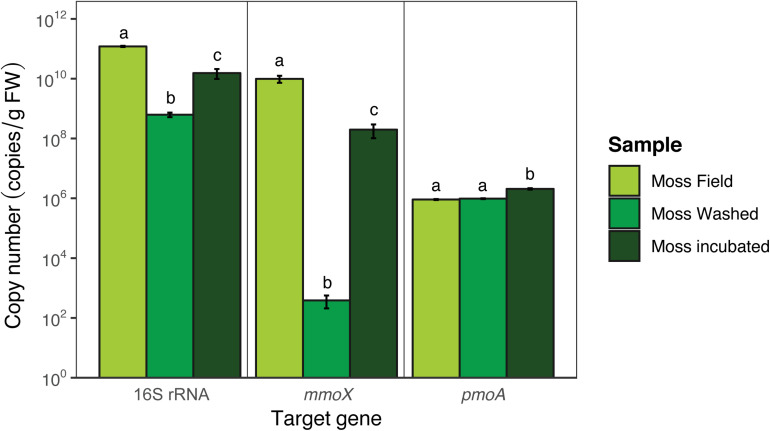
Copy numbers of bacterial 16S rRNA, *pmoA*, and *mmoX* genes obtained via qPCR. Error bars indicate the standard error of the mean (*n* = 3). For each target gene the significant differences between the different samples are indicated by the use of different letters, similar letters indicate no difference.

To quantify the microbial community, qPCR and amplicon sequencing of 16S rRNA genes was performed. Quantification of the bacteria per gram of FW (16S rRNA gene; Figure 5) showed that bacterial copy numbers differed between moss from the field and between moss before and after incubation [*F*_(__2_,_6__)_ = 34.3, *p* < 0.001]. 98% of presumably loosely attached microorganisms were removed by washing the moss (Tukey HSD *p* < 0.001). The washing step reduced the abundance of the *mmoX*-containing methanotrophs from 10^10^ to 10^2^ copies per g FW (Tukey HSD *p* < 0.001), whereas *pmoA-*containing methanotrophs were much less affected (remained around 10^5^ copies per g FW; Tukey HSD *p* > 0.05). At the end of the incubation time the copy numbers were 97% of the original value (Tukey HSD *p* < 0.05), indicating regrowth of microorganisms during the incubation in the mesocosm. Quantification of methanotrophic microorganisms by using qPCR targeting the *mmoX* and *pmoA* genes showed a similar trend [*mmoX F*_(__2_,_6__)_ = 40.7, *p* < 0.001; *pmoA F*_(__2_,_6__)_ = 27.1, *p* < 0.001; Figure 5], although *pmoA-*containing methanotrophs were overall less abundant than *mmoX-*containing methanotrophs (resp. 10^6^ vs. 10^10^ copies per g FW). Upon mesocosm incubation *mmoX* copies increased from 10^2^ to 10^8^ (Tukey HSD *p* < 0.001), while *pmoA*-containing methanotrophs marginally increased in copy number per g FW (Tukey HSD *p* < 0.01).

**FIGURE 6:**
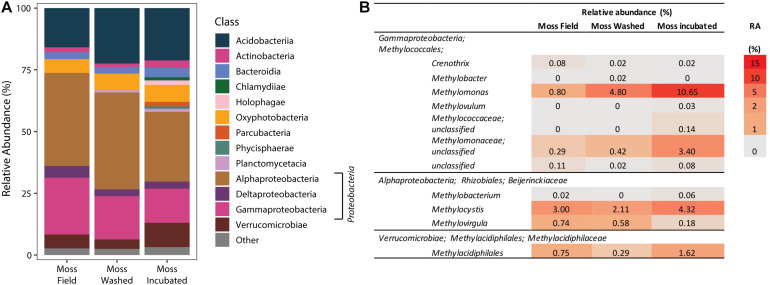
The phylogenetic classification of the bacterial community based on 16S rRNA gene amplification and sequencing **(A)**. Taxonomic groups with a relative abundance >1% are depicted as “other.” In **(B)**, specific relative abundances (RA in %) of methanotrophic bacteria in the bacterial 16S rRNA community profile are shown.

The microbial community composition associated with the mosses was studied by 16S rRNA gene sequencing of the V3–V4 region. Comparison of the moss microbial community in the field and of the community after washing and incubation in the mesocosm showed a gradual change in microbial community (Figure 6 and Supplementary Figure 4). However, the main classes of microorganisms remained the same throughout the incubation. Furthermore, mesocosm incubation increased the microbial community diversity (Shannon and Chao 1 index, Supplementary Table 4), where *Proteobacteria* was the most dominant phylum (Figure 6A). The relative abundance of *Proteobacteria* was not affected by washing, but their relative number increased during incubation in our mesocosm set-up. Furthermore, especially the relative abundance of *Pedosphaerales* and *Opitutales* increased upon incubation (Supplementary Table 5). When focusing on the methanotrophic community, the relative abundance of Verrucomicrobial *Methylacidiphilales* associated to the moss increased after incubation (Figure 6B). Also other methanotrophic bacteria species, such as *Methylomonas* spp. and *Methylocystis* spp., increased in relative abundance upon incubation (Figure 6B) indicating that methane oxidation is facilitated by a number of different methanotrophs.

## Discussion

### *Sphagnum* in Rewetted Peatland Is a Strong Natural CH_4_ Filter

This study aimed to investigate the mitigation of CH_4_ fluxes in rewetted peatlands by an active, natural biofilter composed of *Sphagnum* mosses and their associated CH_4_ oxidizing microorganisms. In our study site, CH_4_ emission was reduced with 93% by *Sphagnum* associated methanotrophs (Figure 2). The reduction of methane emission to the atmosphere by *Sphagnum*-associated methanotrophs could be mimicked in our newly developed mesocosm setup, although the methane emission reduction was less pronounced (31 vs. 93% in the field). Free-floating plants can reduce CH_4_ emission by up to 70% by a combination of plant-associated CH_4_ oxidation and decreased flux rates (Kosten et al., 2016). Also in our mesocosm setup, a dense layer of gnotobiotic *Sphagnum* moss already decreased methane emission, most probably by limiting CH_4_ diffusion from the surface water to the atmosphere. This results in an increase in CH_4_ concentration in the porewater, creating ideal conditions for enrichment of CH_4_ oxidizing microorganisms. All in all, the large decrease of CH_4_ emission in the presence of both submerged *Sphagnum* moss and methanotrophs emphasizes their important role in CH_4_ cycling in peatlands (Basiliko et al., 2004; Kip et al., 2010; Liebner et al., 2011; van Winden et al., 2012, 2020). The tight association between CH_4_ oxidizers and *Sphagnum* mosses is further underlined by the fact that washing of the moss and filtering of the peat water had little effect on CH_4_ oxidation activity.

### *Sphagnum*: A Niche for CH_4_-Oxidizing Microorganisms

QPCR revealed that total bacterial copy numbers per g FW decreased after moss washing. The number of sMMO-containing methanotrophs decreased most, indicating that these methanotrophs might be loosely attached epiphytes. However, subsequently they showed the highest increase in copy number (10^2^–10^8^ copies per g FW) upon mesocosm incubation, which indicates that these microorganisms have a very short generation time. The transcription of *mmoX* gene and activity of sMMO-containing methanotrophs has previously been reported in peatlands (Morris et al., 2002; Chen et al., 2008; Liebner and Svenning, 2012), and together with our findings this suggests that sMMO-containing methanotrophs are relevant for acidic peatland ecosystems.

Surprisingly, the pMMO-containing methanotrophs were initially less abundant compared to sMMO-containing methanotrophs and seemed more tightly associated to *Sphagnum* moss as washing did not decrease their copy numbers. However, there was hardly any increase in abundance of pMMO-containing methanotrophs upon incubation, which might be explained by the lack of copper in our mesocosm incubations (Murrell et al., 2000). Ultimately, the enrichment of sMMO-containing methanotrophs in the mesocosm incubation shows that this set-up can be used to further study the functioning of sMMO methanotrophs in *Sphagnum* mosses. Obtaining more insights into their functioning is of great importance since their ecology is less well understood than that of pMMO-containing methanotrophs.

### *Sphagnum*-Associated Microbial Community

The *Sphagnum-*associated microbial community in all samples showed high similarity to previous *Sphagnum-*associated 16S rRNA gene libraries (Bragina et al., 2012, 2014; Kox et al., 2018). The dominant community members found in this study were similar to those in other investigation with dominant phyla being the *Proteobacteria* (*Alpha*- and *Gammaproteobacteria*), *Cyanobacteria* (*Oxyphotobacteria*) and *Acidobacteria* and a relatively high abundance of *Verrucomicrobia*. Upon mesocosm incubation the microbial diversity increased. The relative abundance of *Verrucomicrobia* and *Planctomycetes* increased, whereas the relative abundance of *Proteobacteria* decreased. Future studies with (micro-) nutrient additions may help to find out what causes these changes in microbial community.

The methanotrophic microbial community profile showed that *Methylacidiphilales, Methylocystis*, and *Methylomonas* spp. all were more abundant at the end the mesocosm incubation. The qPCR profiles showed that the abundance of sMMO containing methanotrophs increased most during incubation. Compared to the 16S rRNA gene library, there are few methanotrophs identified known to possess sMMO. The Verrucomicrobial methanotrophic genera *Methylacidiphilum* and *Methylacidimicrobium* appear to contain only pMMO (Op den Camp et al., 2018), whereas *Methylocystis* species typically have solely pMMO, except for the acidophilic *Methylocystis* isolates *Methylocystis bryophila*, and *Methylocystis heyeri* (Dedysh et al., 2007; Belova et al., 2013) that contain both sMMO and pMMO. *Methylocella* species, facultative methane oxidizers, are the only known organisms containing exclusively sMMO (Dunfield and Dedysh, 2014; Dedysh and Dunfield, 2018). The lack of correlation found in the quantification of *pmmo* and *smmo* genes suggests that sMMO-only microorganisms not belonging to *Methylocella* species are present in our samples. Alternatively, the lack of sMMO-containing methanotrophs in the sequencing analysis could be caused by the coarse taxonomic resolution of the 16S rRNA genes. The presence of sMMO-containing methanotrophs belonging to *Beijerinckiaceae* can thus not be entirely excluded.

### Mesocosm Approach

Studying the *Sphagnum* microbiome in the field is challenging, because the microbial community associated with the moss is influenced by many biotic and abiotic factors which strongly fluctuate in a natural environment. Therefore, we designed a novel mesocosm set-up to mimic a submerged *Sphagnum* moss ecosystem and operated it under controlled laboratory conditions. The conditions could be even more controlled by supplying methane and air continuously to avoid fluctuations in the concentration of these gasses. This would most probably also reduce the fluctuations observed in methane fluxes (Figure 4). We hypothesized that the submerged *Sphagnum cuspidatum* moss layer acts as a biofilter for CH_4_ and expected that the CH_4_-oxidizing microbial community was mainly associated with *Sphagnum* moss. Similar to mosses in the field, results of our controlled mesocosm set-up showed a significant reduction (31%) in CH_4_ emission that was associated with Sphagnum mosses and their microbial community (Figure 4 and Supplementary Figure 8). This CH_4_ removal was only associated with the mosses; methane oxidation activity was not found in the peat water. However, this water can still contain low numbers of methanotrophs. It has been shown before that peat water can be a potential source for methanotrophs which can colonize *Sphagnum* moss (Putkinen et al., 2012).

During incubation in the novel mesocosm set-up, methanotrophic activity indeed increased along with an increase in MOB abundance. CH_4_ oxidation batch-assays revealed a significant increase in methanotrophic activity after incubation (from 121 ± 4 to 189 ± 6 μmol CH_4_ g^–1^ DW day^–1^, resp. Figure 3 and Table 1), indicating MOB involvement in CH_4_ mitigation. Similarly, qPCR of functional methanotrophic genes (*mmoX* and *pmoA*), indicated that significant numbers of CH_4_-oxidizing bacteria were present in and on the moss and that their numbers increased over the course of the incubation.

The reduction in CH_4_ emission in the mesocosm set-up was lower than the reduction found in the field, which is most likely due to peat moss density that is much higher in the field (∼50 cm deep in de field compared to 6 cm in the mesocosm). Mesocosm incubations were terminated after 32 days; we believe that the CH_4_ mitigation by the moss-associated methanotrophs in the mesocosm will increase even further by prolonging the incubation time. In addition, an increased *Sphagnum* moss density is expected to increase the CH_4_ oxidation even further. Furthermore, the mesocosm set-up could be improved by a continuous supply system for CH_4_ and air, which results in a system that is more comparable to the natural situation.

### Implications for Degraded Peatlands

The large organic matter stocks in peatlands are a potential source for CO_2_. Restoration measures aimed at preventing CO_2_ emission often involve hydrological measures (rewetting; Lamers et al., 2002; Smolders et al., 2003), which result in high CH_4_ production rates (Abdalla et al., 2016). Since peatland degradation affects the presence and abundance of *Sphagnum* (Gorham, 1991; Frolking et al., 2011), care should be taken to bring back and facilitate *Sphagnum* mosses in restored peatlands. Stimulation of the current population or even reintroduction of *Sphagnum* in peatland restoration projects can thereby strongly mitigate the resultant CH_4_ emissions.

## Conclusion

*Sphagnum* mosses have many key roles in peat ecosystems (Rydin and Jeglum, 2006), and our study shows that their microbiome and specifically their associated methanotrophs are crucial to reduce CH_4_ emissions from peatlands. Peatland restoration practices involving rewetting typically result in high CH_4_ emissions and should therefore simultaneously aim to stimulate the presence of *Sphagnum* mosses. With the development of our mesocosm setup, CH_4_ mitigation by *Sphagnum* mosses and their associated methanotrophs can be studied in great detail, providing essential knowledge that can be used for restoration practices and climate research in the future.

## Data Availability Statement

The datasets presented in this study can be found in online repositories. The names of the repository/repositories and accession number(s) can be found below: https://www.ncbi.nlm.nih.gov/genbank/, PRJNA517391.

## Author Contributions

MARK, AJPS, and MAHJvK collected the samples. MARK and MAHJvK processed the samples and wrote the manuscript with input from all authors. MARK, DRS, MSMJ, and MAHJvK designed the research. MARK performed the experiments. HJMOdC, MSMJ, LPML, and AJPS were involved in project discussion and data interpretation. All authors contributed to the article and approved the submitted version.

## Conflict of Interest

The authors declare that the research was conducted in the absence of any commercial or financial relationships that could be construed as a potential conflict of interest.
