# Supplementary material for: A Novel Laboratory-Scale Mesocosm Setup to Study Methane Emission Mitigation by Sphagnum Mosses and Associated Methanotrophs
Source: Front Microbiol. 2021 Apr 26;12:652486. doi: 10.3389/fmicb.2021.651103 (PMC8108401; doi:10.3389/fmicb.2021.651103)
Supplement: Supplementary file 1 [file Data_Sheet_1.PDF]

Supporting information

Table S1 Change in read count during data processing.

Step		Moss Field Read count (average)		SEM	Moss Washed Read count (average)		SEM	Moss incubated Read count (average)		SEM
1	Unprocessed reads	31 301	±	2 569	32 001	±	2 117	45 380	±	13 738
2	Screen sequences	28 443	±	2 351	29 082	±	1 958	41 585	±	12 791
3	Unique sequences	27 912	±	2 311	28 488	±	1 923	40 541	±	12 439
4	Aligned sequences	27 912	±	2 311	28 488	±	1 923	40 541	±	12 439
5	Screened alignment	27 912	±	2 311	28 488	±	1 923	40 541	±	12 439
6	Filter positions in alignment	27 912	±	2 311	28 488	±	1 923	40 541	±	12 439
7	Pre-clustering	27 912	±	2 311	28 488	±	1 923	40 541	±	12 439
8	Chimera removal	23 924	±	2 032	24 129	±	1 667	31 278	±	9 097
9	Non-target lineage removal	7 847	±	578	9 028	±	669	18 003	±	5 700
	Total read count per group	*23 542*			*27 083*			*54 009*		
10	Random subsample	6 500	±	0	6 500	±	0	6 500	±	0

Table S2 Change in read quality during data processing.

Step		Total read count	# Unique reads	Mean read length (bp)	Max. ambiguent positions	Max. polymers
1	Unprocessed reads	326 045		444	308	297
2	Screen sequences	297 333		445	0	45
3	Unique sequences	297 333	154 224	445	0	45
4	Align sequences	297 333	154 224	407	0	28
5	Screen alignment	290 823	148 011	407	0	8
6	Filter positions in alignment	290 823	82 822	407	0	8
7	Pre-clustering	290 823	50 548	407	0	8
8	Chimera removal	237 994	12 021	407	0	8
9	Non-target lineage removal	104 634	10 068	411	0	8

Table S3 Primer sequences used in this study.

Target gene	Primer name	Sequence	Reference
pmoA	A189f	5'-GGNGACTGGGACTTCTGG-3'	(Holmes et al. 1995)
	A682r	5'-GAASGCNGAGAAGAASGC-3'	(Holmes et al. 1995)
mmoX	mmoX1	5'-CGGTCCGCTGTGGAAGGGCATGAAGCGCGT-3'	(Miquez et al. 1997)
	mmoX2	5'-GGCTCGACCTTGAACTTGGAGCCATACTCG-3'	(Miquez et al. 1997)
16S rRNA	Bact 341f	5′-CCTACGGGNGGCWGCAG-3′	(Klindworth et al. 2013)
Bacteria	Bact 785r	5'-GACTACHVGGGTATCTAATCC-3	(Klindworth et al. 2013)

Table S4 Alpha diversity index for the different samples.

Sample	Observed species number (S)	Richness				Evenness
		Shannon (H)	Fisher's alpha	Chao1	ACE	Pielou (J)
**Moss Field**	1136	4.53	263.0	1607.3	1729.4	0.64
**Moss Washed**	1215	4.23	287.0	1762.1	2001.5	0.60
**Moss Incubated**	1213	4.77	286.4	2046.3	2061.7	0.67

Tables S5 Relative abundances (RA in %) of Verrucomicrobial microorganisms in the bacterial 16s rRNA community profile.

	Relative abundance (%)		
	Moss Field	Moss Washed	Moss incubated
Verrucomicrobia; Verrucomicrobiae;			
Chthoniobacterales	0.42	0.25	0.20
Methylacidiphilales	0.29	0.12	0.44
Opitutales	0.11	0.13	0.36
Pedosphaerales	0.53	0.34	1.23
S-BQ2-57_soil_group	0.00	0.01	0.00
unclassified	0.02	0.03	0.04
Verrucomicrobiales	0.01	0.01	0.08

RA (%)
1.6
0.8
0.4
0.2
0.1
0

Table S6 Mean potential CH_4 oxidation rate in mosses (μmol CH_4 g^{-1} DW day^{-1}) and peat water (μmol CH_4 ml^{-1} day^{-1}) prior to mesocosm incubation round 1.

Round 1					
**Material**	**Treatment**	**Methane oxidation rate** (μmol CH_4 g^{-1} DW day^{-1})	**SE**	**R2**	**n**
**Moss**	Washed	121	4.1	0.98	3
**Moss**	Field	165	29	0.99	3
**Material**	**Treatment**	**Methane oxidation rate** (μmol CH_4 ml^{-1} day^{-1})	**SE**	**R2**	**n**
**Water**	Filtered	0.01	0.003	0.40	3
**Water**	Field	0.08	0.02	0.48	3

Table S7 Mean potential CH_4 oxidation rate in mosses (µmol CH_4 g^{-1} DW day^{-1}) and peat water (µmol CH_4 ml^{-1} day^{-1}) prior to mesocosm incubation round 2.

Round 2					
**Material**	**Treatment**	**Methane oxidation rate** (µmol CH_4 g^{-1} DW day^{-1})	**SE**	**R2**	**n**
**Moss**	Washed	215	7.6	0.99	3
**Moss**	Field	281	14	0.99	3
**Material**	**Treatment**	**Methane oxidation rate** (µmol CH_4 ml^{-1} day^{-1})	**SE**	**R2**	**n**
**Water**	Filtered	-0.04	0.03	0.35	3

Table S8 Mean potential CH_4 oxidation rate in mosses (µmol CH_4 g^{-1} DW day^{-1}) and peat water (µmol CH_4 ml^{-1} day^{-1}) after mesocosm incubation round 2.

Round 2						
**Material**	**Mesocosm**	**Treatment**	**Methane oxidation rate** (µmol CH_4 g^{-1} DW day^{-1})	**SE**	**R2**	**n**
**Moss**	Moss		121	12	0.97	3
**Moss**	Moss	+ inhibitor	-2.0	0.6	0.33	3
**Material**	**Mesocosm**	**Treatment**	**Methane oxidation rate** (µmol CH_4 ml^{-1} day^{-1})	**SE**	**R2**	**n**
**Water**	Moss		0.10	0.02	0.94	3
**Water**	Moss	+ inhibitor	-0.02	0.00	0.71	3
**Water**	Control		-0.01	0.01	0.39	3
**Water**	Control	+ inhibitor	-0.02	0.00	0.91	3

Table S9 Elemental composition of the unfiltered and filtered porewater.

porewater	NO_3^- (µmol L^{-1})	NH_4^+ (µmol L^{-1})	PO_4^{3-} (µmol L^{-1})	Al (µmol L^{-1})	Ca (µmol L^{-1})	Fe (µmol L^{-1})	K (µmol L^{-1})
unfiltered	1.22 ± 0.07	12.58 ± 2.83	0.68 ± 0.05	9.27 ± 2.21	30.51 ± 1.06	14.69 ± 2.45	9.03 ± 0
filtered	2.17 ± 0.54	3.55 ± 0.27	0.31 ± 0.06	4.52 ± 0.36	33.25 ± 2.06	4.12 ± 0.17	5.18 ± 0

porewater	Mg (µmol L^{-1})	Mn (µmol L^{-1})	Na (µmol L^{-1})	P (µmol L^{-1})	S (µmol L^{-1})	Si (µmol L^{-1})	Zn (µmol L^{-1})
filtered	26.62 ± 0.67	0.44 ± 0.02	172.44 ± 3.32	2.04 ± 0.21	24.08 ± 0.58	9.45 ± 0.40	0.58 ± 0
unfiltered	21.70 ± 0.04	0.46 ± 0.02	170.04 ± 3.28	0.48 ± 0.12	20.61 ± 0.67	9.85 ± 0.21	0.43 ± 0

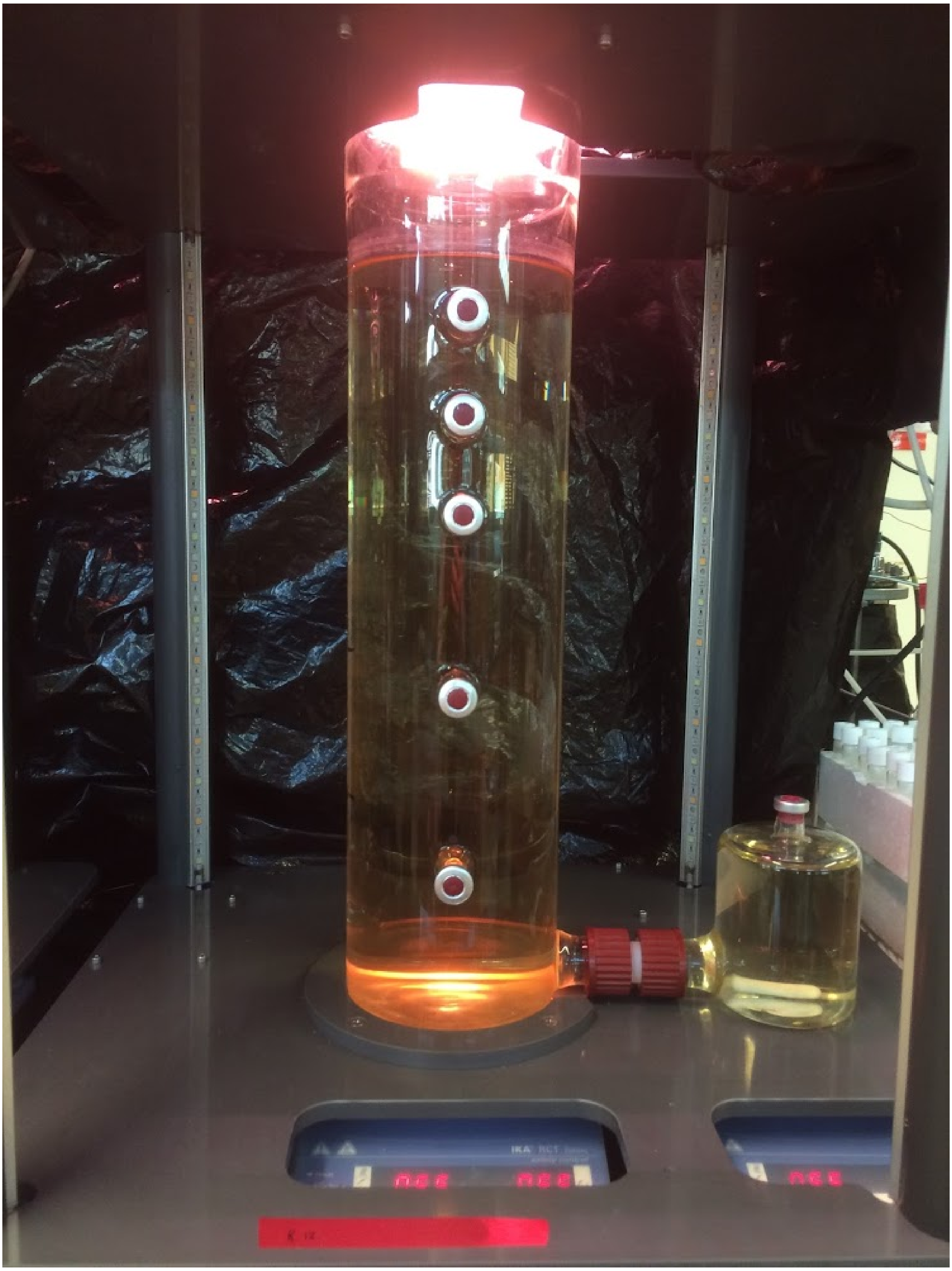

Fig. S1 Photo of the real mesocosm column set-up.

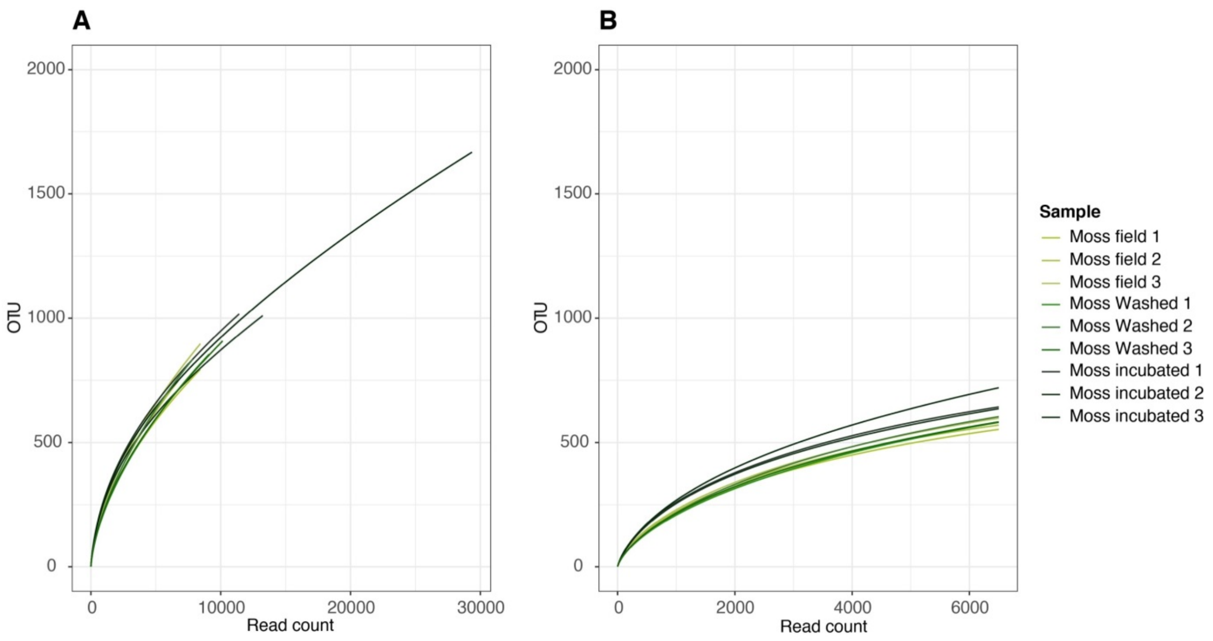

Fig. S2 Rarefaction curves before **(A)** and after **(B)** subsampling, singletons were removed.

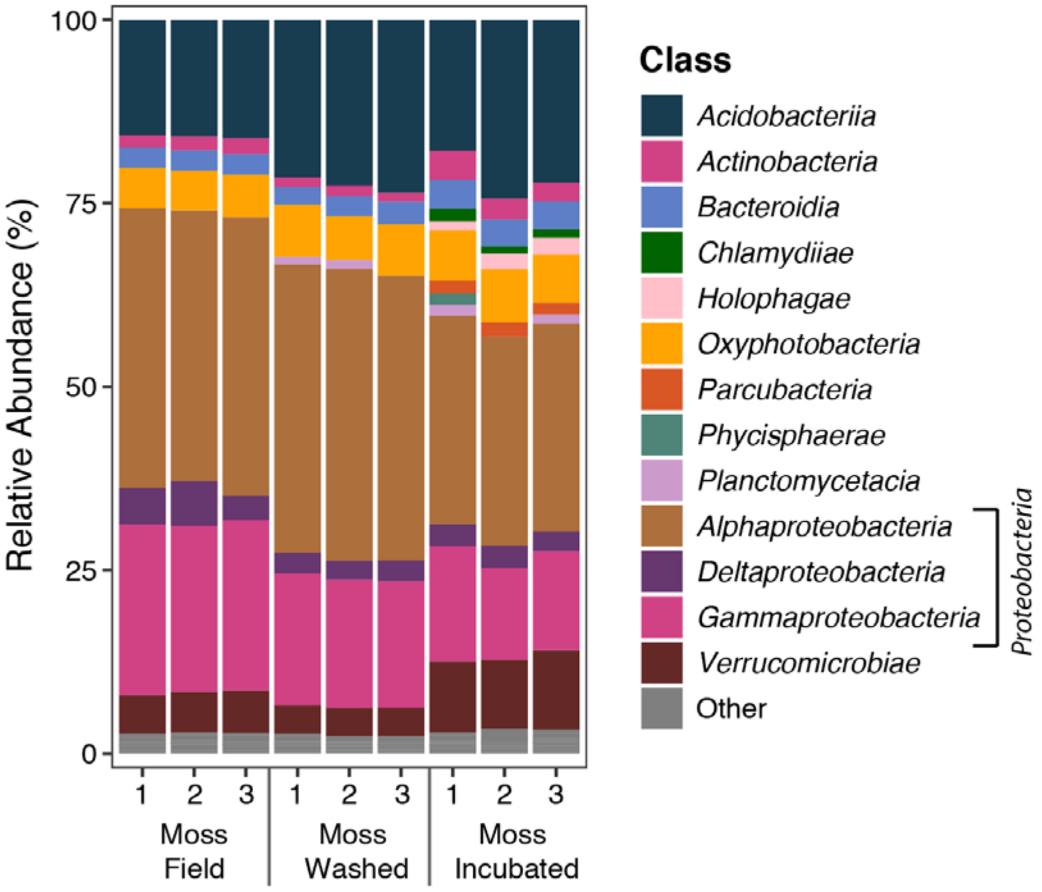

Fig. S3 Phylogenetic classification of the bacterial community per sample based on 16s rRNA gene amplification and sequencing. Taxonomic groups with a relative abundance <1% are depicted as "Other".

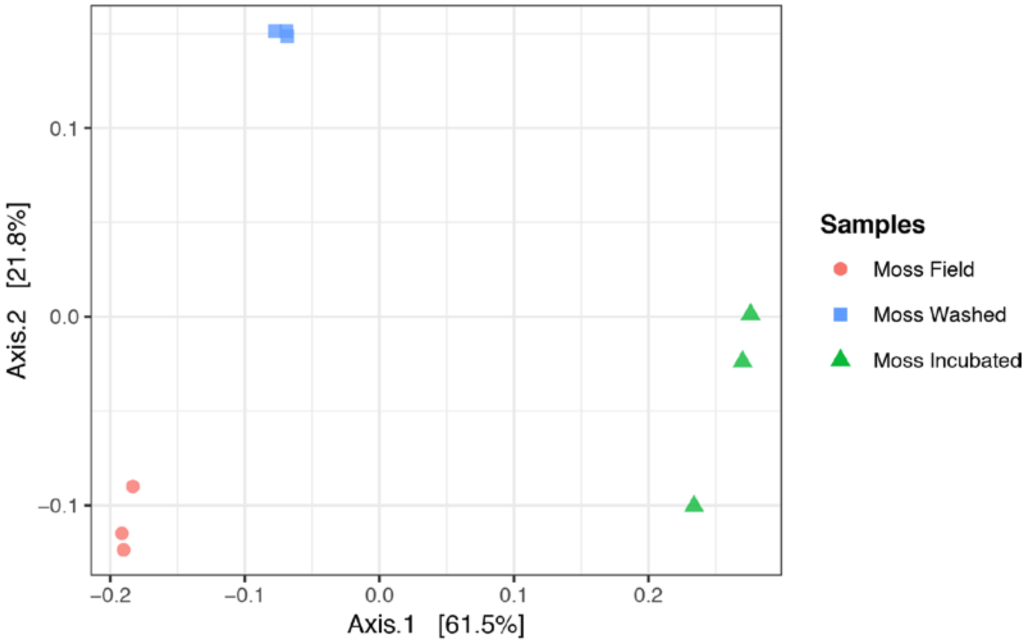

Fig. S4 Principal Coordinate Analysis (PCoA) of the microbial diversity using Bray Curtis dissimilarity. The different samples are coded according to colour.

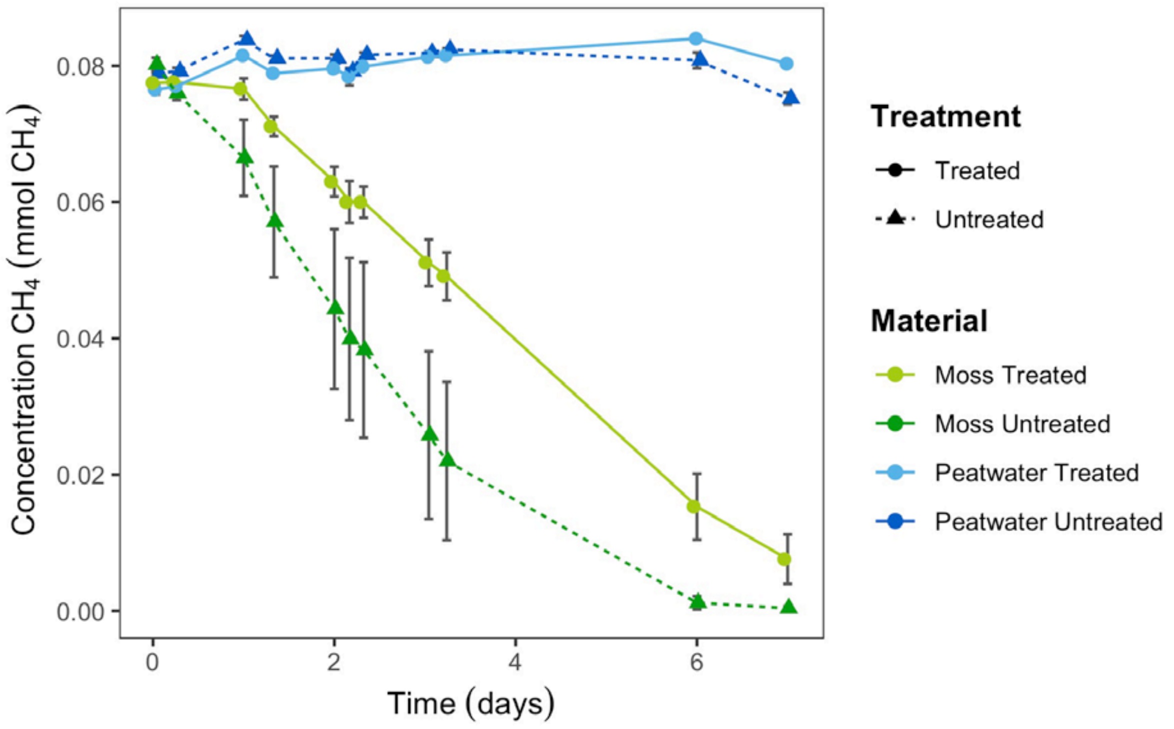

Fig. S5 Potential CH_4 oxidation rate in batch assays, prior to mesocosm incubation round 1. CH_4 concentration (mmol) in the headspace is displayed over time (days) for incubations with: mosses (unwashed (dark green, n=3) and washed (light green, n=3)) and peat water (unfiltered (dark blue, n=3) and filtered (light blue, n=3)). Error bars indicate the standard error of the mean.

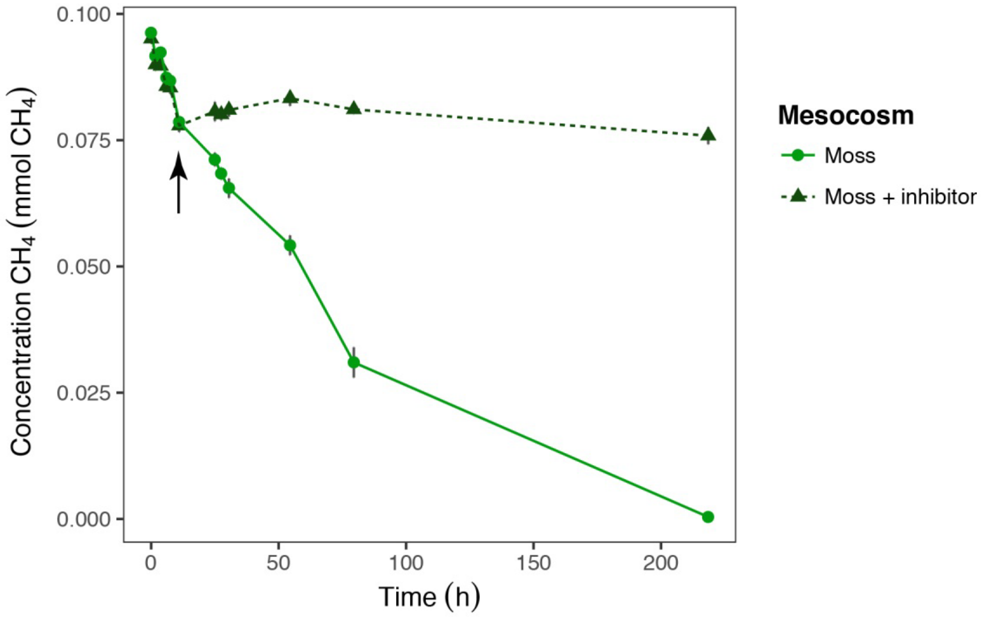

Fig. S6 Potential CH_4 oxidation rate of the mosses in batch assays, after mesocosm incubation round 1. CH_4 concentration (mmol) in the headspace is displayed over time (h) for incubations with mosses, either with PMO inhibitor acetylene (light green, n=3) or without PMO inhibitor (dark green, n=3). Arrow indicates addition of acetylene gas. Error bars indicate the standard error of the mean.

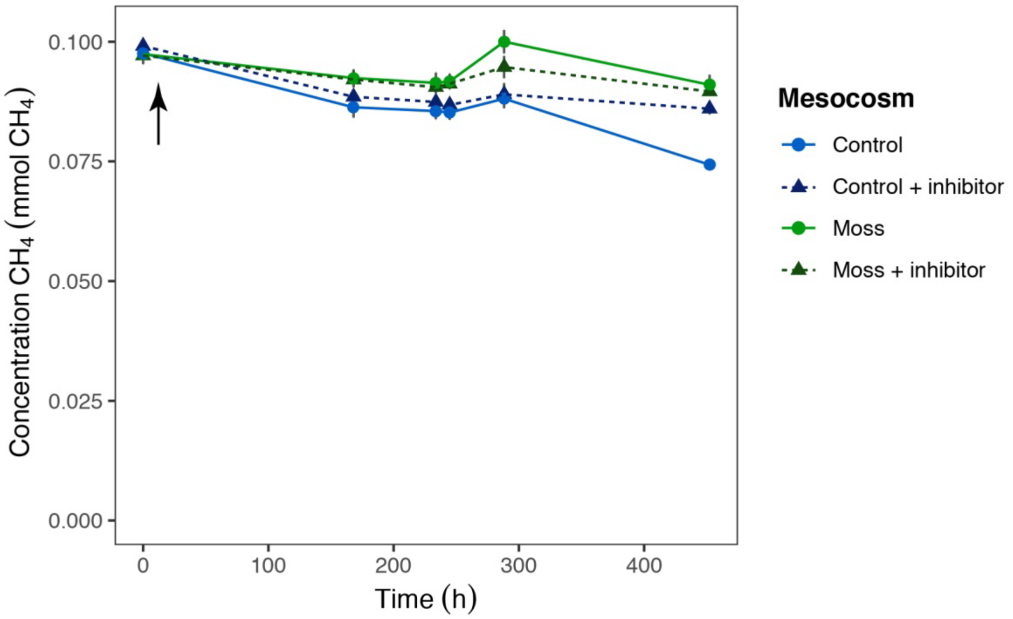

Fig. S7 Potential CH_4 oxidation rate of peat water in batch assays, after mesocosm incubation round 1. CH_4 concentration (mmol) in the headspace is displayed over time (h) for incubations with peat water from the moss mesocosm (green) or peat water from the control mesocosm (blue). Incubations are either with PMO inhibitor (lighter colors, dashed line, n=3) or without PMO inhibitor (darker colors, dashed line, n=3). Arrow indicates addition of acetylene gas. Error bars indicate the standard error of the mean.

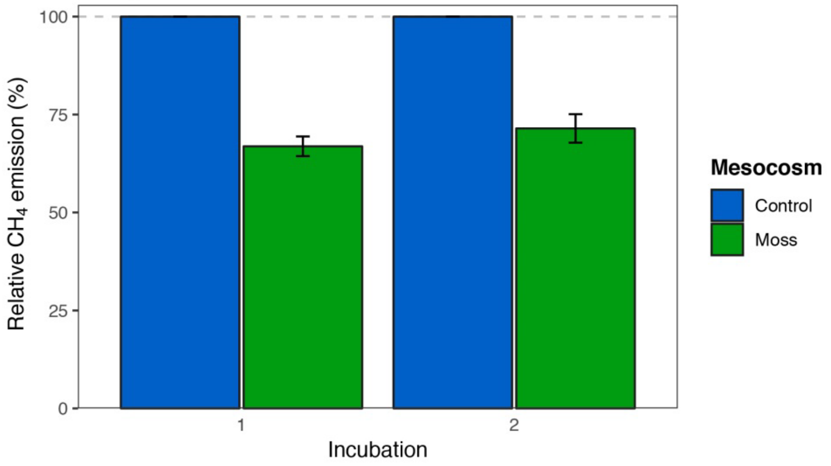

Fig. S8 Relative CH_4 emission (as % of emission in control mesocosm) from the control mesocosm (blue) and the moss mesocosms (green), shown for incubation round 1 and incubation round 2. Each bar represents the average relative CH_4 emission of days 8-32 and error bars indicate the standard error.

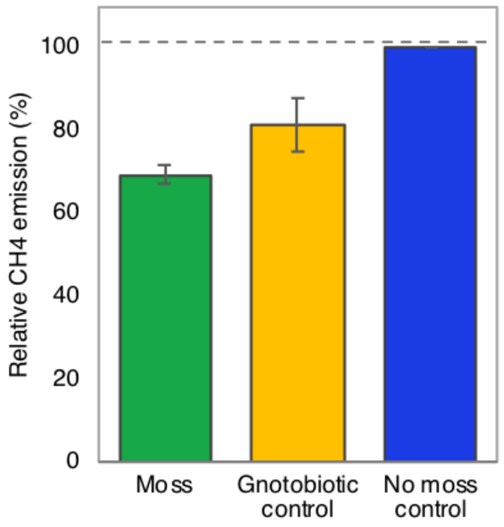

Fig. S9 Relative CH_4 emission (as % of emission in control mesocosm) from the mesocosms with moss from the field (green) and the control mesocosms with gnotobiotic moss (yellow) or without moss (no moss (blue). Each bar represents the average relative CH_4 emission of days 8-32 and error bars indicate the standard error.
